# Efficient Activation of Peroxymonosulfate by V-Doped Graphitic Carbon Nitride for Organic Contamination Remediation

**DOI:** 10.3390/ma15248936

**Published:** 2022-12-14

**Authors:** Zhi Deng, Zhenhua Huang, Jun Liu, Yongkui Huang, Peili Lu

**Affiliations:** 1 Key Laboratory of Shale Gas Exploration, Ministry of Natural Resources, Chongqing Institute of Geology and Mineral Resources, Chongqing 401120, China; 2 State Key Laboratory of Coal Mine Disaster Dynamics and Control, College of Environment and Ecology, Chongqing University, Chongqing 400044, China

**Keywords:** g-C_3_N_4_, advanced oxidation processes, photocatalysis, peroxymonosulfate, organic contamination

## Abstract

Advanced oxidation processes (AOPs) based on peroxymonosulfate (PMS) activation have been developed as an ideal pathway for completely eradication of recalcitrant organic pollutants from water environment. Herein, the V-doped graphitic carbon nitride (g-C_3_N_4_) is rationally fabricated by one-step thermal polymerization method to activate PMS for contamination decontamination. The results demonstrate the V atoms are successfully integrated into the framework of g-C_3_N_4_, which can effectively improve light absorption intensity and enhance charge separation. The V-doped g-C_3_N_4_ displays superior catalytic performance for PMS activation. Moreover, the doping content has a great influence on the activation performances. The radical quenching experiments confirm •O_2_^−^, SO_4_^•−^, and *h*^+^ are the significant species in the catalytic reaction. This work would provide a feasible strategy to exploit efficient g-C_3_N_4_-based material for PMS activation.

## 1. Introduction

The organic contamination in groundwater and wastewater has become a challenging issue in the development of a sustainable society [1,2,3]. A number of toxic and persistent organic pollutants such as endocrine disrupting chemicals, antibiotics, and petroleum hydrocarbon, are frequently found in groundwater [4,5,6]. These pollutants are difficult to be biodegraded because of their chemical stability and environmental risk [7,8]. To date, various treatment techniques have been developed for organic contamination remediation, such as membrane separation, anaerobic technologies, and adsorption [3,5]. Unfortunately, their application are dramatically impeded by the low removal efficiency [3,9,10]. It is imperative but still remains challenges to develop cost-effective treatment methods for addressing organic contamination issues.

In recent years, advanced oxidation processes (AOPs) have been developed as an ideal pathway for completely eradication of recalcitrant organic pollutants from water environment [3,9,11]. In this process, the structure of organic contaminants can be destroyed by the highly reactive oxygen species [3,12,13]. Among various active radicals, sulfate radicals (SO_4_^•−^) generated from peroxymonosulfate (PMS) activation have been widely exploited due to their stronger oxidative capacity and longer lifetime [14,15,16]. More importantly, SO_4_^•−^ is generally reacted with target compounds via an electron transfer pathway due to its high selectivity [12,17]. Therefore, many approaches have been extensively applied to activate PMS into SO_4_^•−^, such as electrochemistry, transition metal ions, UV, and photocatalysis [18,19]. It is recognized that the photocatalysis is a promising tool for PMS activation owing to its low cost and environmentally compatible [20,21,22]. Nevertheless, it is still a big challenge to explore advanced materials with high quantum efficiency and abundant active sites to meet the practical requirement for PMS activation.

Recently, a large variety of novel materials have been widely used in PMS activation, including semiconductor composites, perovskite, and carbon-based materials [3,11,13,23]. Among them, graphitic carbon nitride (g-C_3_N_4_) has been demonstrated as an effective heterogeneous catalyst due to its good stability and facile synthesis [24,25]. Especially, g-C_3_N_4_ possesses unique optical and electronic properties (ca. 2.7 eV), indicating that it can be excited by visible light with a wavelength of less than 460 nm [26,27]. Nevertheless, the major drawbacks of pure g-C_3_N_4_ are the poor visible light response, sluggish charge separation efficiency and low surface area [25,28]. Various strategies have been employed to overcome these drawbacks, including morphological control, vacancies, heteroatom doping, and the construction of heterojunctions [24]. Among these methods, heteroatom doping into the frameworks carbon has become a pivotal strategy to develop g-C_3_N_4_-based photocatalysts with pronounced catalytic performance [29,30,31,32]. In principle, element V may be a promising dopants to boost catalytic performance of g-C_3_N_4_ due to its comparable atom size. Wang et al., reported that V doped g-C_3_N_4_ catalysts shows an enhanced performance in the catalytic dehydrogenation of propane [33]. Neelakanta Reddy et al., reported that the incorporation of V into the host g-C_3_N_4_ material can enhance the catalytic activities in water splitting [32]. Yet, the utility of V-doped g-C_3_N_4_ for PMS activation has never been investigated.

Herein, the V-doped g-C_3_N_4_ are rationally fabricated by the thermal polymerization method. The structure, morphology, and optical properties were characterized using various analysis technologies, such as X-ray diffraction (XRD), Fourier transform infrared (FT-IR), X-ray photoelectron spectroscopy (XPS), scanning electron microscopy (SEM), and UV-vis diffuse reflectance spectroscopy spectra (DRS). The bisphenol A (BPA), as an emerging recalcitrant contaminant, was selected to systematically probe the performance of the V-doped g-C_3_N_4_. The doping V atoms could effectively increase light absorption intensity and enhance charge separation. As expected, the V-doped g-C_3_N_4_ displays superior catalytic performance for PMS activation. Finally, a possible activation mechanism was discussed and proposed based on the capture experiment and photoelectrochemical measurement.

## 2. Materials and Methods

### 2.1. Chemicals

Melamine, ammonium vanadate (NH_4_VO_3_), p-benzoquinone (BQ) and ethylenediaminetetraacetic acid dihydrate (EDTA) were purchased from Aladdin Industrial Co., Shanghai, China. Nafion solution (5.0 wt%), peroxymonosulfate (PMS, KHSO_5_·0.5KHSO_4_·0.5K_2_SO_4_), and bisphenol A (BPA) were purchased from Sigma-Aldrich Co., LTD (St. Louis, MO, USA). Sodium sulfate (Na_2_SO_4_), sodium hydroxide (NaOH), isopropanol (IPA), and methanol (MeOH) were obtained from Sinopharm Chemical Reagent Co., Ltd. (Shanghai, China). All chemical reagents are of analytical grade and used without further purification. Deionized (DI) water with a resistivity of 18.2 MΩ was used throughout the experiments.

### 2.2. Synthesis of Materials

The V-doped g-C_3_N_4_ was synthesized via the thermal polymerization method. In a typical procedure, 5 g of melamine was thoroughly ground with an appropriate amount of NH_4_VO_3_. The collected mixture was placed into a crucible with a cover, and calcined at 550 °C for 4 h. The product was washed with NaOH solution (1 M), ethanol and water, and dried at 60 °C. A series of V-doped g-C_3_N_4_ were obtained by adjusting the weight ratio of NH_4_VO_3_ and melamine (0.1, 0.2, 0.5, and 0.8%) and denoted as V-g-C_3_N_4_-0.1, V-g-C_3_N_4_-0.2, V-g-C_3_N_4_-0.5, and V-g-C_3_N_4_-0.8.

### 2.3. Materials Characterization

The TEM (Talos F200S microscope) and SEM (JSM-7800F microscope, Tokyo, Japan) were conducted to study the morphology. The XRD (PANalytical Powder X-ray diffractometer), FT-IR (Thermo Ni-colet iS 50, Waltham, MA, USA), and XPS spectroscopy (Thermo ESCA LAB 250, Waltham, MA, USA) were measured to analyze the surface elemental compositions. The DRS was recorded on a Shimadzu UV-3600 (Shimadzu, Kyoto, Japan). The N_2_ adsorption−desorption measurements were performed on an N_2_ adsorption apparatus (Micromeritics ASAP 2020, Norcross, GA, USA).

### 2.4. Activation Performance Tests

The photodegradation of BPA was selected as a model reaction to study the activation performance of the V-doped g-C_3_N_4_. In a typical catalytic experiment, the sample (50 mg) was put in 50 mL BPA solution (10 mg/L), and stirred in the dark for 30 min. The 300 W Xe lamp with a 400 nm cut-off filter (CEL-HXF300, Aulight, Beijing, China) was immediately turned on after a specific amount of PMS was added. The experiments were conducted at 25 °C. The pH was not controlled during the experiment. At specified time intervals, the sample was taken out from the solution and filtered with a PTFE filter. Then, 0.1 mL of methanol was added immediately to quench radicals. The amount of BPA was analyzed by high performance liquid chromatography (HPLC, Agilent 1260, Santa Clara, CA, USA) with an Agilent C-18 column and UV detection wavelength of 276 nm. The mixture solutions of 30% water and 70% methanol were used as the mobile phase, and the flow rate was 1.0 mL‧min^−1^.

For the quenching experiment, BQ, EDTA, IPA, and MeOH were added as quenchers for different radicals.

### 2.5. Electrochemical Measurements

The Mott-Schottky plots and electrochemical impedance spectroscopy (EIS) were obtained on a CHI-660E electrochemical analyzer (CH Instruments, Shanghai, China) in a standard three electrode configuration. The Ag/AgCl and Pt sheet were used as the reference electrode and the counter electrode, respectively. The working electrode was prepared on indium doped tin oxide (ITO) substrates. The 1 mg sample was dispersed in a mixture containing 0.98 mL of anhydrous ethanol and 0.02 mL of Nafion solution by ultrasonication for 30 min. The obtained slurry was dropped onto the surface of the ITO glass with a size of 1 × 1 cm^2^, and then dried at room temperature. 0.5 M Na_2_SO_4_ was employed as the electrolyte. The Mott-Schottky plot was obtained through measurement of the capacitance as a function of potentials at 1 kHz. The EIS measurements were carried out in the frequency range of 10^−2^ to 10^5^ Hz with a 10 mV amplitude.

## 3. Results and Discussion

### 3.1. Characterizations of Materials

**Figure 1:**
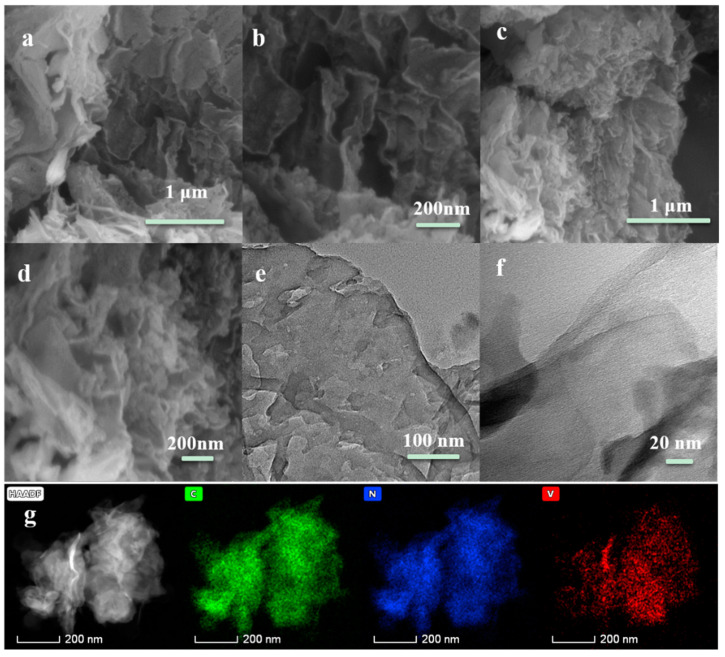
SEM of g-C_3_N_4_ (**a**,**b**) and V-g-C_3_N_4_-0.5 (**c**,**d**); TEM (**e**), HR-TEM (**f**) and Elemental mappings (**g**) of V-g-C_3_N_4_-0.5.

The structure and morphology of the g-C_3_N_4_ and V-g-C_3_N_4_-0.5 samples were characterized by SEM and TEM. From Figure 1a, pure g-C_3_N_4_ shows a typical 2D nanosheet-stacked structure with a micrometer scale lateral size. Figure 1b further confirms that the 2D sheet structure has a loose and flats surface. Figure 1c,d reveals the V-g-C_3_N_4_-0.5 displays a similar layered structure and morphology with that of g-C_3_N_4_. TEM was further utilized to reveal the detailed structure of V-doped g-C_3_N_4_. The layer sheet morphology with some porous structure is clearly observed in V-g-C_3_N_4_-0.5 (Figure 1e), which may be generated by the release of gas during the thermal decomposition of precursors [34,35,36]. The HRTEM image (Figure 1f) reveals that no crystalline phases are seen on the surface of g-C_3_N_4_, which is consistent with the results of SEM [31,37]. The homogeneous distribution of N, C, and V elements on the entire sample are shown in the element mapping (Figure 1g). Thus, the V atoms are successfully introduced into the framework of g-C_3_N_4_.

**Figure 2:**
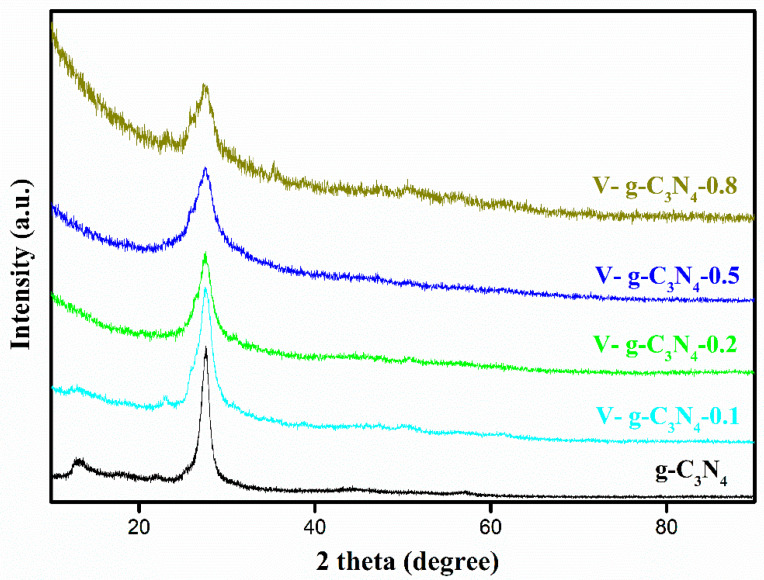
XRD patterns of the materials.

The chemical structures of the materials were investigated by XRD patterns. As shown in Figure 2, the g-C_3_N_4_ shows two distinct peaks at 12.8 and 27.6°, corresponding to the extended distance of the interplanar repeat unit in the (1 0 0) diffraction plane and the stacking of the π-π conjugated aromatic rings in the (0 0 2) diffraction plane, which is in accordance with previous studies [29,37,38]. The peak related to the (0 0 2) facets of g-C_3_N_4_ is clearly noticed in V-doped g-C_3_N_4_. However, the weaker diffraction peak at 12.8° has become indistinct gradually with increasing ratios of NH_4_VO_3_, suggesting its low crystallinity and disordered structure. Interestingly, no peaks of V oxides are found in V-doped g-C_3_N_4_. Thus, the V atoms are integrated into the framework of g-C_3_N_4_.

**Figure 3:**
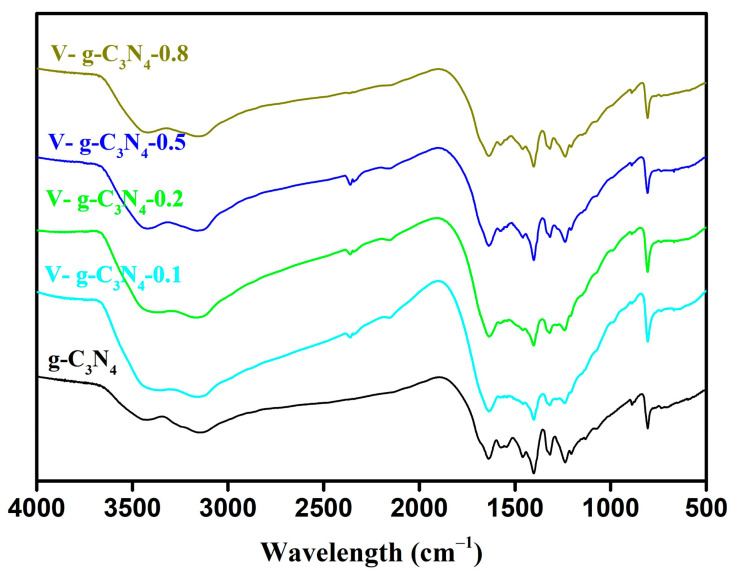
FT-IR spectra of the materials.

The FT-IR spectra of the synthesized materials are illustrated in Figure 3. The absorption bands at 1200~1700 cm^−1^ are attributed to the stretching of the C-N heterocycle [27,39]. Specifically, the peaks positioned at 1314 and 1238 cm^−1^ are corresponded to the stretching vibrations of the connected units of N−(C)_3_ and C−NH−C, respectively. The broad band ranging from 3000 to 3650 cm^−1^ is assigned to the stretching vibrations of surface hydroxyl groups (OH-) and terminal and residual amino-groups (NH_2_-and NH-) [31,40]. The strong peak of 808 cm^−1^ is corresponded to the bending mode of C−N heterocycles [41,42]. Comparatively, the FT-IR spectra of the V-doped g-C_3_N_4_ are similar to that of pure g-C_3_N_4_. In addition, no characteristic absorption peaks of V-O are detected, which can be due to the low content of V atoms. These findings confirm the doping V atoms does not significantly alter the main structure and functional groups of g-C_3_N_4_.

**Figure 4:**
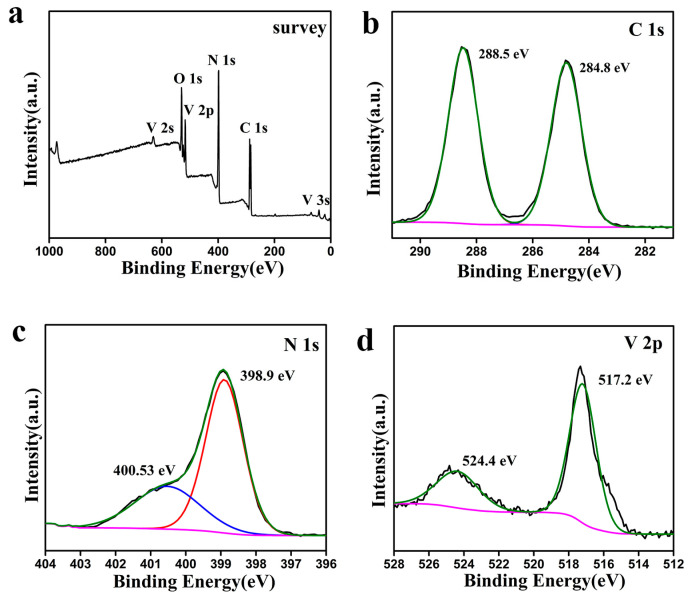
XPS spectroscopy of V-g-C_3_N_4_-0.5: survey spectra (**a**), C 1s (**b**), N 1s (**c**), and V 2p (**d**).

The valence states of the V-doped g-C_3_N_4_ sample were further identified by XPS. The XPS survey spectra (Figure 4a) prove the coexistence of N, C, and V elements in the V-g-C_3_N_4_-0.5 sample. The C 1s spectrum in Figure 4b exhibits two characteristic peaks at 288.5 and 284.8 eV, ascribing to the sp2 hybridized carbon (N−C=N) and graphitic carbons (C=C), respectively [38,42]. The N 1s spectrum in Figure 4c shows two peaks at binding energies of 398.9 and 400.5 eV, which are ascribed to the hybridized aromatic N atoms bound to C atoms in the triazine units (C−N=C) and tertiary nitrogen (N-(C)_3_, respectively [37,38,43]. Furthermore, two major peaks at binding energies of 517.2 and 524.4 eV are observed from the V 2p spectrum (Figure 4d), which are belonged to the V 2p_3/2_ and V 2p_1/2_ of V-O bond of V^5+^ species, respectively [34]. These results further confirm the V atoms are successfully implanted in the framework of the g-C_3_N_4_.

**Figure 5:**
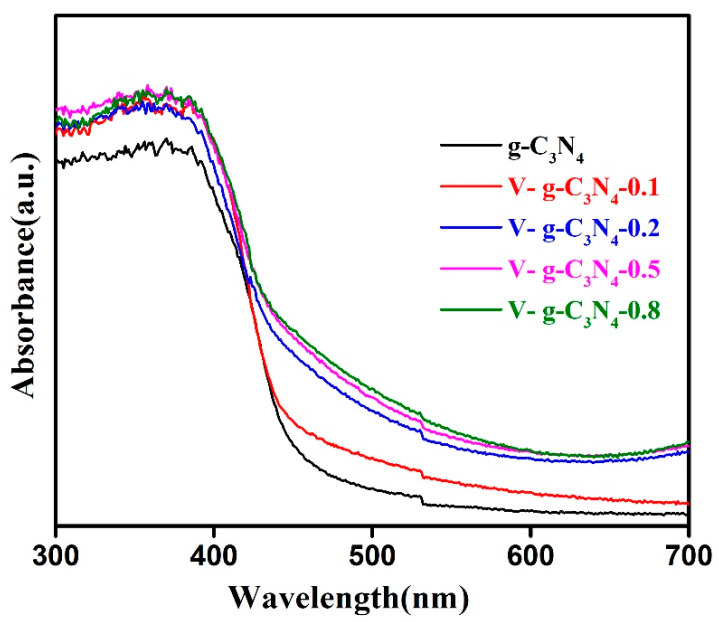
DRS spectra of the samples.

The DRS was performed to gain insights into the influence of V doping on the optical property of g-C_3_N_4_. As shown in Figure 5, the g-C_3_N_4_ possesses a distinct visible-light absorption feature with an absorption edge of about 450 nm, which is consistent with previous studies [28,43]. As observed, the V-doped g-C_3_N_4_ samples show wide and strong absorption in the visible light range. Moreover, a slight redshift of the absorption edge can be observed for the V-doped g-C_3_N_4_ compared with that of g-C_3_N_4_. The visible-light absorption abilities of V-doped g-C_3_N_4_ enhances gradually with increasing the dopant concentration, revealing superior visible light utilization [29,44]. Therefore, the doping V atoms could effectively strengthen light absorption intensity, which would boost the catalytic performance of g-C_3_N_4_.

### 3.2. Activation Performance

**Figure 6:**
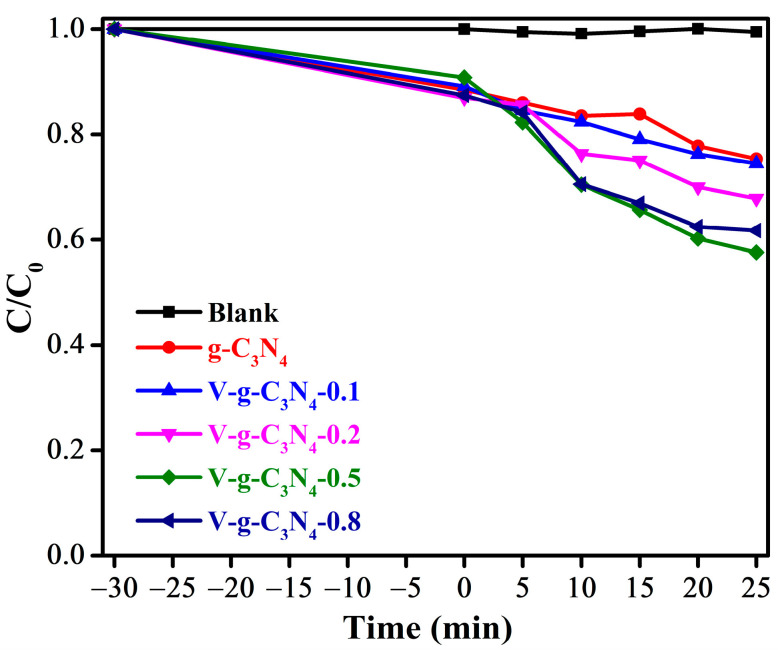
The removal efficiency of BPA by the prepared samples.

The BPA was selected as the target organic pollutant to preliminarily probe the catalytic properties of the V-doped g-C_3_N_4_. As shown in Figure 6, the BPA concentration remains almost the same without any catalyst, suggestting that BPA is stable under visible light irradiation. Only 24.7% of BPA is removed by pure g-C_3_N_4_, indicating that the catalytic capability of g-C_3_N_4_ is considerably low. This result is similar to the previous studies [29,31]. Obviously, the V-doped g-C_3_N_4_ samples show relatively high catalytic activity compared with g-C_3_N_4_, which can be assigned to the doping V atoms into the networks of g-C_3_N_4_. Moreover, the catalytic capability of V-doped g-C_3_N_4_ are firstly increased as the V content increased. When the content of V is further increased, the degradation efficiency of BPA is slightly decreased. These results manifest that the dopant content plays a crucial role in the catalytic capability of V-doped g-C_3_N_4_.

**Figure 7:**
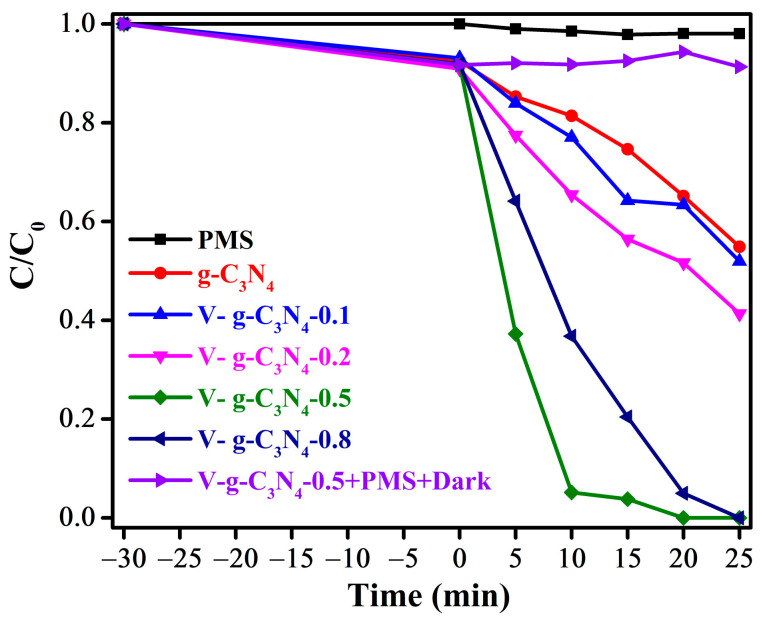
Removal efficiency of BPA by the prepared samples in the presence of PMS.

The PMS activation performance of the prepared samples for organic pollutions degradation was further verified. As shown in Figure 7, the negligible removal of BPA is observed with the addition of PMS, suggesting the oxidation of PMS can be negligible. The pure g-C_3_N_4_ shows obvious catalytic activity in PMS activation under visible light irradiation for BPA degradation, owing to its unique electronic structure and moderate band gap. Furthermore, the V-doped g-C_3_N_4_ possesses excellent catalytic performance in PMS activation. Meanwhile, the catalytic performance of the V-doped g-C_3_N_4_ is increased firstly and then decreased with the increase of doping content, in which the BPA can be completely removed within 25 min over V-g-C_3_N_4_-0.5. In addition, the control experiment illustrates the visible light irradiation also plays the key role in PMS activation for organic pollutions degradation. It should be pointed out that the trend is consistent with the photocatalytic degradation of BPA in absence of PMS, further confirming the catalytic performances for PMS activation comes from the intrinsic photocatalytic capability of V-doped g-C_3_N_4_. This could be explained by the fact that the doping V atoms in the networks of g-C_3_N_4_ would decrease band gap energy and improve carrier utilization, resulting in the enhanced degradation efficiency. Nevertheless, an excess of V atoms might conduct recombination sites for charge carriers, which resulted in abatement of catalytic performance. The results illustrate that the doping content has great effects on the catalytic performances of V-doped g-C_3_N_4_, and the V-g-C_3_N_4_-0.5 has the best performance.

**Figure 8:**
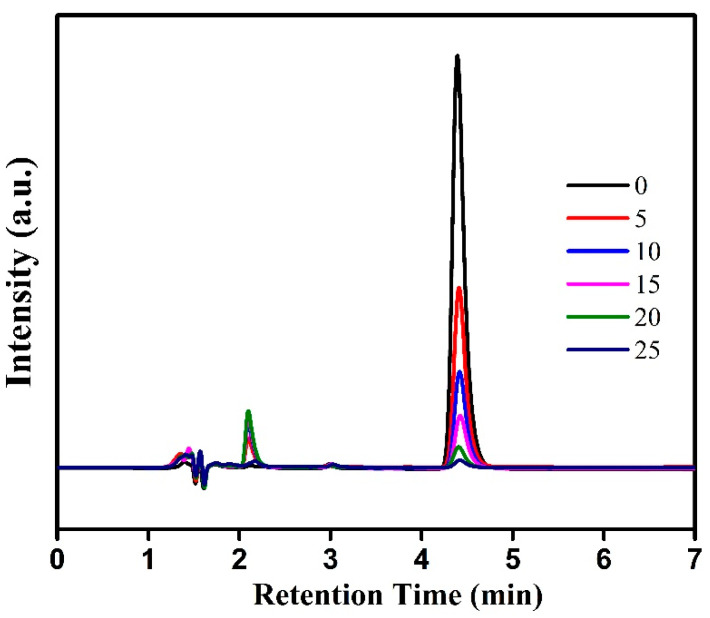
HPLC spectra of BPA degradation over the V-g-C_3_N_4_-0.5 with PMS activation.

In order to further clarify the performance of V-doped g-C_3_N_4_ for catalytic activation of PMS, HPLC was used to study the degradation process of BPA. As shown in Figure 8, the peak of BPA is declining with the increase of reaction time, and disappeared after 25 min. A weak peak is increased and then dropped during the reaction process, which might be ascribed to the generation intermediates from the degradation reaction of BPA. Furthermore, it is worth noting that this peak is disappeared completely after the completion of the catalytic reaction. These results indicates that BPA can be successfully mineralized to CO_2_ and H_2_O. The similar result is also observed in previous literatures [22,45]. In addition, this system was also compared with previously reported literatures, and the results are listed in Table 1. These result reveal that the V-doped g-C_3_N_4_ performs superior catalytic capability for PMS activation under visible light.

### 3.3. Activation Mechanism

**Figure 9:**
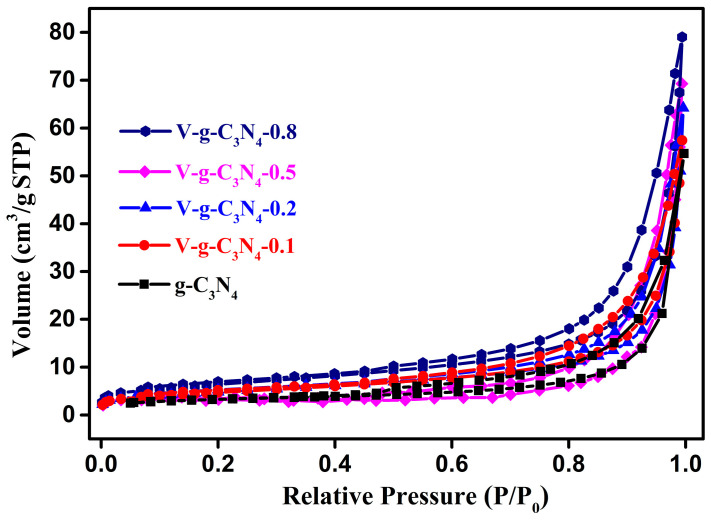
N_2_ sorption isotherms of the g-C_3_N_4_ and V-doped g-C_3_N_4_ samples.

The surface area and microstructures of the g-C_3_N_4_ and V-doped g-C_3_N_4_ samples were determined by the N_2_ sorption isotherms measurement. As presented in Figure 9, all of them show a similar shape of type IV isotherms and H2 hysteresis loops, indicating the presence of some mesopores. The specific surface areas of the g-C_3_N_4_, V-g-C_3_N_4_-0.1, V-g-C_3_N_4_-0.2, V-g-C_3_N_4_-0.5, and V-g-C_3_N_4_-0.8 were calculated to be ca. 16.95, 17.63, 19.69, 22.63, and 25.56 m^2^/g, respectively. It can be seen that the doping V atoms into the framework of g-C_3_N_4_ may slightly ameliorate the specific surface areas. Thus, the specific surface areas has little impact on the catalytic activity.

**Figure 10:**
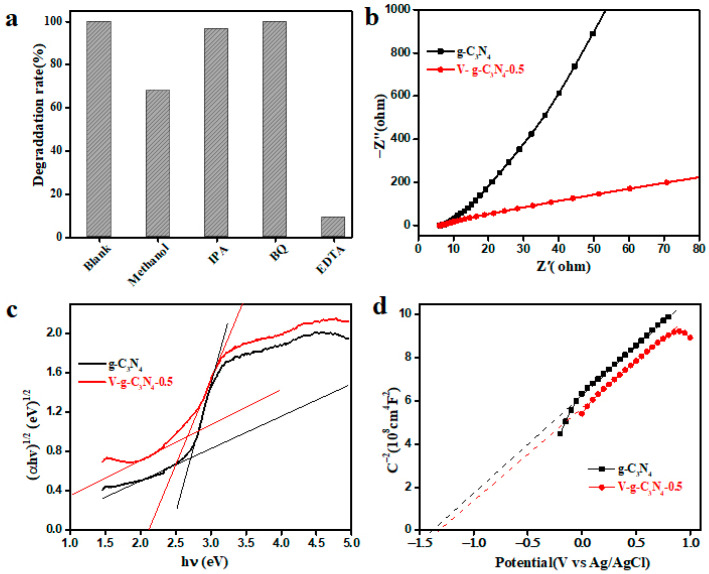
The results of reactive species scavengers (**a**); EIS curves (**b**), Tauc plots (**c**), and Mott-Schottky curves (**d**).

For an in-depth investigation of the degradation mechanism, the quenching experiments were performed to identify the reactive species in this catalytic reaction system (Figure 10a). After the addition of BQ and EDTA, the degradation rate is remarkably decreased, implying the •O_2_^−^ and *h*^+^·have pivotal roles in BPA degradation [20,21]. However, the degradation rate is not obviously changed by the addition of IPA, suggesting •OH plays a minor role in this degradation reaction. Meanwhile, the degradation reaction of BPA is markedly inhibited by MeOH, indicating that the SO_4_^•−^ plays a significant role. Therefore, •O_2_^−^, SO_4_^•−^, and *h*^+^ are the important species in the catalytic destruction of BPA under the visible light.

In principle, the charge transfer exhibits a positive relationship with the catalytic performance of PMS activation [51]. Thus, the separation efficiency of generated carriers over V-g-C_3_N_4_-0.5 and g-C_3_N_4_ was investigated by EIS. From the EIS Nyquist plot (Figure 10b), the V-g-C_3_N_4_-0.5 shows a smaller semi-circular radius than pure g-C_3_N_4_, indicating a high charge mobility and a low interfacial resistance. Therefore, the doping V atoms can effectively ameliorate the electronic conductivity, enhance charge separation, and decrease the charge carrier recombination rate.

The electronic structure of prepared samples was further explored by the Tauc plot and Mott-Schottky analysis to understand the charge transfer mechanism [30,52,53,54]. It is widely known that the band gap energy (Eg) of semiconductors can be estimated according to the Kubelka-Munk method [52,55,56,57]. As illustrated in Figure 10c, the Eg of g-C_3_N_4_ and V-g-C_3_N_4_-0.5 are determined as 2.72 and 2.66 eV, respectively. This result reveals that doping V atoms can narrow the band gap of g-C_3_N_4_. Furthermore, the CB potential of g-C_3_N_4_ and V-g-C_3_N_4_-0.5 were analyzed from Mott-Schottky curves. As exhibited in Figure 10d, the g-C_3_N_4_ and V-g-C_3_N_4_-0.5 shows a positive slope, verifying they are n-type semiconductors. Meanwhile, the flat-band potentials of g-C_3_N_4_ and V-g-C_3_N_4_-0.5 are −1.38 and −1.31 V (Ag/AgCl, pH = 7). Accordingly, the CB position of g-C_3_N_4_ and V-g-C_3_N_4_-0.5 are determined to be −1.16 and −1.09 eV (NHE, pH = 7), respectively. Furthermore, the VB position of g-C_3_N_4_ and V-g-C_3_N_4_-0.5 are 1.56 and 1.57 eV (NHE, pH = 7), indicating that the doping V atoms has no significant influence on the VB position of g-C_3_N_4_.

**Figure 11:**
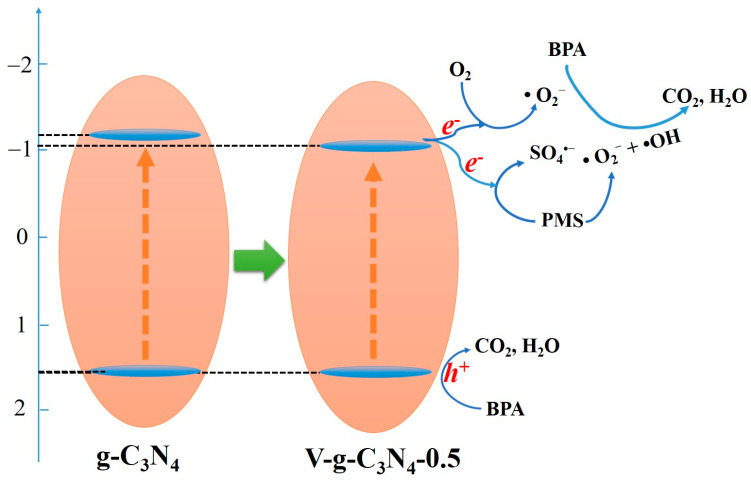
The proposed catalytic mechanism of PMS activation on the V-doped g-C_3_N_4_.

Based on the above results, a schematic diagram of the mechanism for the PMS activation over the V-doped g-C_3_N_4_ under light irradiation is proposed in Figure 11. The V-doped g-C_3_N_4_ are excited to produce electrons and holes by visible light irradiation. These electrons and holes would shift onto the VB and CB edges, respectively. Afterwards, these excited electrons can activate PMS to generate SO_4_^•−^. Additionally, these photoinduced electrons would capture O_2_ molecules to generate •O_2_^−^. The *h*^+^ accumulated on the CB edges possesses strong oxidation activity to mineralize organic compounds. It should be noted that the •OH cannot be directly formed by oxidization of *h*^+^ due to the thermodynamic limitations [29,34]. Besides, the •OH could be formed via interconversion reactions of •O_2_^−^ and SO_4_^•−^ [15,37]. Therefore, the BPA molecules can be mineralized under the synergistic effect of •O_2_^−^, SO_4_^•−^, *h*^+^, and •OH.

## 4. Conclusions

In summary, the V-doped g-C_3_N_4_ was successfully designed and fabricated via one-step thermal polymerization method to activate PMS toward organic contamination degradation. The results reveal that the V atoms are successfully integrated into the structure of g-C_3_N_4_, which can effectively increase visible light absorption intensity and enhance charge separation. The obtained V-doped g-C_3_N_4_ shows superior catalytic performance for PMS activation, in which the BPA can be completely removed within 25 min. Moreover, doping content has a great influence on the activation performances. The radical quenching experiments confirm the •O_2_^−^, SO_4_^•−^, and *h*^+^ are significant species in degradation reactions. Finally, a possible degradation mechanism is discussed and proposed. This work provides a feasible strategy to optimize the performance of novel materials for environmental remediation.

## Figures and Tables

**Table 1:** A comparison of the catalytic performance with other works.

Catalysts	BPA (mg/L)	Catalyst Dosages (g/L)	PMS (mM)	Removal Time (min)	Removal Rates (%)	Ref.
CNT-TiO_2_	10	0.5	1.7	40	90	[46]
WO_3_@MoS_2_/Ag	10	0.8	3.3	140	92.5	[23]
cobalt-to-oxygen doped g-C_3_N_4_	15	0.4	-	210	100	[47]
PI-g-C_3_N_4_	10	1	5	60	100	[48]
CQDs/g-C_3_N_4_	20	0.33	0.1	30	95	[49]
ZIF-NC/g-C_3_N_4_	20	/	1	60	97	[50]
C-O modified g-C_3_N_4_	10	1	1.5	36	100	[45]
V-doped g-C_3_N_4_	10	1	1	25	100	This work

## Data Availability

Data are contained within the article.
